# New Drugs, Appliances, and Things Medical

**Published:** 1890-09-06

**Authors:** 


### NEW DRUGS, APPLIANCES, AND THINGS MEDICAL.

[All preparations, appliances, novelties, etc., of which a notice is desired, should be sent for The Editor, to care of The Manager, 140, Strand, London, W.C.]

### PETER MÖLLER'S COD LIVER OIL.

We have received a specimen of this old and well-known article. It is a beautifully clear yellow, bright, and clean looking oil and it answers to all the usual tests. Its taste is to those who can digest cod liver oil far from unpleasant. When a man has taken a dozen gold medals with his manufactures, there is usually little left to be said in praise of them, and all we need to add is that Möller's oil is as good as ever. We know of none better. By-the-bye we may mention that many children will take the oil very well in mashed potatoes, more especially if the potatoes are accompanied by fish of any kind. Most children get so accustomed to the taste and even to like the oil that they are always ready for it when the time comes round for the dose. There are some who can never manage it either pure, which is the best form, emulsionized by pancreatic ferments or alkalies, or in any of the various flavoured and disguised methods now so commonly employed. There are one or two rules about keeping cod liver oil that should be remembered. 1. Don't put it on a shelf over a hot fire-place, or in full sunlight. The oil very quickly alters and oxidises, becoming like old train oil in appearance, smell and taste, and so most disgusting to the unfortunate patients or children who have to take it. Keep it in a cool dark cupboard or larder. 2. Always wipe the mouth of the bottle clean, also the glass from which the oil is taken, for the same reason as above, because the oil is oxidised by the air and so becomes unpleasant. It is no uncommon thing to see the oily glass standing beside the bottle, ready for the next dose. A dirty and disgusting habit!

### MOSELY'S "COMPACTA" CABINETS FOR MICRO-SLIDES.

The patentee of the above has sent us a specimen. The advantage claimed for it, is that by one motion all the slides in the cabinet have their labels exposed at once, so that each tray has not to be lifted off in the search for a particular specimen. We think Mr. Mosely has succeeded admirably in effecting his object and has certainly shortened the toil of the hard worked microscopist. The cabinets are to be obtained of most dealers in such requisites and are made in the the usual sizes.

### "BONTHA."

A pleasant aërated drink stated to be made from tea and free from alcohol and excess of tannin. Our examination bears this statement out. We think a slight degree more of flavouring would be an improvement. It may be obtained from May, Davis, and Co., Westminster Bridge Road.

### BOVININE

is the name of a fluid preparation of beef prepared in Chicago by a cold process. It is stated to contain as much as from 18 to 20 per cent. of coagulable albumen. The profession in America have for some time made use of it in cases of gastric irritability and of extreme exhaustion. It seems from its form to be of very considerable usefulness for rectal alimentation. It should be given cold, or only slightly warmed, as heat coagulates it readily, also not with fluids containing tannin or strong alcohol for the same reason. We have examined the sample sent us. It is a dark red, nearly clear, fluid. Under the microscope are to be seen some fat globules of small size, considerable quantities of altered blood cells, and granular mattter. On testing it by heat, nitric acid, and absolute alcohol, a large coagulum was formed. Further tests bear out the analysis given in the pamphlets accompanying the sample. We think Bovinine likely to fill a useful place among the forces for combating the wasting effects of fever in any form.

A house physician has ventured on a little grumble, and pointed out to the nurses of the London Hospital that he has no salary, no regular hours of duty, and is liable to be called up in the middle of the night. Perhaps there are no class of men who perform so much unremunerated labour as doctors; during their time of training as house physician or surgeon this is all very well, but a visiting surgeon once counted up that he spent eight hundred hours, or just one hundred working days, at his honorary duties at the hospital, and also attended one hundred private patients who, on the ground of being dependent on professional brethren, claimed gratuitous treatment. When the public point their jokes at the wealth of some half-dozen of our rich medical men, they forget that the point is blunted to those who know how dearly such positions are won, and how many are the physicians who labour perpetually yet never grow rich.

